# ILC1s in Tissue Inflammation and Infection

**DOI:** 10.3389/fimmu.2016.00104

**Published:** 2016-03-22

**Authors:** Anja Fuchs

**Affiliations:** ^1^Department of Surgery, Washington University School of Medicine, St. Louis, MO, USA

**Keywords:** ILC1, innate lymphoid cells, inflammation, host response, ILC development, antimicrobial defense

## Abstract

Innate lymphoid cells (ILCs) are innate immune cells that provide an early source of cytokines to initiate and tailor the immune response to the type of the encountered pathogen or insult. The group 1 ILCs are comprised of conventional natural killer (cNK) cells and subsets of “unconventional NK cells,” termed ILC1s. Although cNK cells and ILC1s share many features, such as certain phenotypic markers and the ability to produce IFN-γ upon activation, it is now becoming apparent that these two subsets develop from different progenitors and show unique tissue distribution and functional characteristics. Recent studies have aimed at elucidating the individual contributions of cNK cells and ILC1s during protective host responses as well as during chronic inflammation. This review provides an overview of the current knowledge of the developmental origins as well as of the phenotypic and functional characteristics of ILC1s.

## Introduction

Innate lymphoid cells (ILCs) are a recently identified group of cells of the innate immune system that modulate immune functions prior to the generation of an adaptive immune response. Unlike T cells and B cells, ILCs lack antigen-specific receptors; instead, their function is mediated by cytokines released by antigen-presenting cells or damaged tissue cells in response to infection or tissue damage. Activated ILCs secrete cytokines that tailor the immune response to the encountered pathogen or insult. Based on their secreted cytokine profile, ILCs are regarded as the innate equivalent of adaptive T cells: group 1 ILCs are equivalent to Th1 T cells, group 2 ILCs to Th2 T cells, and group 3 ILCs to Th17 and Th22 cells (1–4). The group 1 ILCs are comprised of conventional natural killer (cNK) cells and several groups of “unconventional NK cells,” termed ILC1s, which differ from conventional NK cells in their phenotypes, locations, functions, and/or transcription factor dependence. Within group 1 ILCs, cNK cells appear to be more potent in mediating cytotoxicity (“killer ILCs”), while the primary role of ILC1s is the production of pro-inflammatory cytokines (“helper-like ILCs”) (2). This review will briefly touch on the key features of conventional NK cells as the prototype of group 1 ILCs and will then discuss in more detail the known characteristics of the “unconventional NK cells,” referred to here as ILC1s. Several subsets of ILC1s have been identified, which are located predominantly within non-lymphoid tissues, such as the intestinal mucosa, liver, salivary gland, and in the female reproductive tract. For some of the ILC1 subsets, a clear lineage relationship to ILC1s or cNK cells has not firmly been established. The paucity of specific surface markers and transcription factors for ILC1s has made studying these cells difficult. The ILC field in general is a relatively young field of study, and within this field, group 1 ILCs are the least-well studied ILCs compared to ILC2s and ILC3s. This review aims at giving an overview of the current knowledge on the phenotype and developmental origin of ILC1 subsets, as well as their roles during antimicrobial immune defense and chronic inflammation.

## Heterogeneity within Group 1 ILCs: Location, Phenotype, and Functions

The unifying characteristic of group 1 ILCs is their ability to produce IFN-γ. Apart from this common attribute, significant differences exist between the known ILC1 subsets. The following sections summarize the current knowledge of the tissue location, surface marker expression, and functional characteristics of the main group 1 ILC subsets.

### Conventional NK Cells

Conventional NK cells are predominantly found as circulating in the blood and within secondary lymphoid tissues such as the lymph nodes and spleen. However, cNK cells are also present in some non-lymphoid tissues such as the liver and the lung. Resting mature cNK cells are identified as CD3ϵ^−^ NK1.1^+^ NKp46^+^ DX5 (CD49b)^+^ lymphocytes in the mouse (Table 1) and as CD3ϵ^−^ CD56^+^ NKp46^+^ NKp44^−^ cells in humans (Table 2) (5, 6). Mature cNK cells express the transcription factors T-bet and Eomes, which are important in mediating their functions. cNK cells can be activated through cytokines, such as IL-12, IL-15, and IL-18, which induces their secretion of IFN-γ and TNF-α. Furthermore, cNK cells are activated through ligation of specific surface receptors. cNK cells express a variety of activating and inhibitory NK cell receptors that allow them to differentiate between self and non-self and between healthy and infected or malignantly transformed cells. Activation of cNK cells through these surface receptors typically triggers cytokine production and can induce cytotoxicity of target cells via cNK cell release of perforin and granzymes. Through these functions, cNK cells fulfill a potent pro-inflammatory role during the host–response to microbial pathogens. In particular, cNK cell-mediated recognition and elimination of altered host cells contributes to antiviral immunity and tumor immunosurveillance (6–10). In humans, two main cNK cell subsets exist that differ in their phenotype and functions: the majority of blood cNK cells are CD56^low^ and display high cytotoxic potential, while the minor CD56^high^ subset of cNK cells has low cytotoxic functions but shows potent production of inflammatory cytokines (10, 11). In contrast to the peripheral blood compartment, CD56^high^ cNK cells represent the major cNK cell subset within human secondary lymphoid tissues such as lymph nodes, tonsils, and spleen (12). In mice, a subset of cNK cells with thymic origin and preferential homing to lymph nodes has been identified that may represent the equivalent of human CD56^high^ cNK cells (13). The lineage relationships and specific functions of these different cNK cell subsets during host–responses are still being investigated. Blurring the lines between innate and adaptive lymphocytes, there is accumulating evidence that demonstrates close parallels between cNK cells and adaptive lymphocytes. Similar to T cells, cNK cells can undergo an expansion and contraction phase during microbial infections, followed by the generation of a pool of memory cells. These memory cNK cells possess enhanced cytolytic and cytokine responses during secondary exposure to certain microbial pathogens, a finding that suggests that immunological memory is not confined to adaptive lymphocytes (14).

**Table 1:** **Surface marker and transcription factor expression by murine group 1 ILCs**.

	NK1.1	NKp46	DX5	TRAIL	CD49a	CD160	CD127	CD11b	CD69	CXCR6	T-bet	Eomes	Reference
Splenic cNK	+	+	+	–	–	–	–	+	–	–	+	+	(6, 17, 25, 37, 45, 47, 59)
LP ILC1	+	+	–	n.d.	+	+	+	–	+	±	+	–	(20, 30, 45, 47)
ieILC1	+	+	–	+	+	+	±	–	+	±	+	+	(2, 19, 45, 47)
Liver ILC1	+	+	–	+	+	+	±	–	+	±	+	–	(16, 17, 20, 25, 26, 45, 47)
Salivary ILC1	+	+	+	+	+	n.d.	–	+	+	n.d.	+	+	(34, 35, 36)
Uterine ILC1	+	+	–	–	+	n.d.	–	n.d.	+	n.d.	+	±	(17, 37, 40)

*±, heterogenous expression; n.d., not determined*.

**Table 2:** **Surface marker and transcription factor expression by human group 1 ILCs**.

	CD56	NKp46	NKp44	CD103	CD49a	CD160	CD127	CD11b	CD69	CD16	CXCR6	T-bet	Eomes	Reference
Blood cNK (CD56^low^)	+	+	–	–	–	–	–	+++	±	+++	–	+	+	(2, 5, 10, 11, 31)
Blood cNK (CD56^high^)	+++	+	–	–	–	–	+	+++	±	–	–	+	+	(2, 10, 11, 31)
LP ILC1	–	–	–	–	n.d.	+	+	n.d.	±	–	n.d.	±	–	(18, 24)
ieILC1	±	+	+	+	+	+	–	–	+	–	+	+	+	(19, 24)
Liver ILC1	+++	+	–	±	±	n.d.	–	n.d.	+	–	+	+	±	(31, 32)

*±, heterogenous expression; n.d., not determined*.

### ILC1s

Unconventional NK cells (ILC1s) are currently defined as tissue-resident NK-like cells that do not develop from conventional NK cell precursors (NKP) and are not typically found in blood or lymphoid organs. Subsets of ILC1s have been identified in a variety of non-lymphoid tissues, including small intestinal mucosa, liver, salivary glands, and the female reproductive tract (Tables 1 and 2). In mice, these pools of tissue-resident ILC1s do not recirculate and appear to be maintained predominantly via local self-renewal rather than through replenishment from blood-derived ILC1s or their precursors (15–17). Apart from these common attributes, significant differences exist between the individual ILC1 subsets in regards to their phenotype and known functions. Furthermore, for some of these subsets, which appear to be ILC1s based on their characteristic cytokine production, a clear lineage relationship to other ILC1s or cNK cells has not firmly been established. For the purpose of this review, I will focus on some of the better-characterized members of the ILC1 lineage, which includes the subsets of ILC1s in the intestine and liver.

### Intestinal ILC1s

Intestinal ILCs that express typical NK cell receptors –NKp46 and NK1.1 in the mouse; NKp46 and/or CD56 in humans – have in the past collectively been considered as cNK cells due to their NK cell-like phenotype and their ability to produce IFN-γ. However, evidence is now emerging that the intestinal mucosa contains several types of group 1 ILCs that are distinct from cNK cells. Two main subsets of intestinal ILC1s were recently identified in mice and humans: intraepithelial ILC1s (ieILC1s) and lamina propria-resident ILC1s (LP ILC1s) (18–20). ieILC1s are found in the intestinal epithelium and resemble cNK cells in their expression of canonical NK cell receptors. However, unlike resting cNK cells, ieILC1s express surface markers typical of intraepithelial T cells, such as the integrin CD49a, the activation marker CD69, the HVEM receptor CD160, and in humans, the integrin CD103 (19). To date, it is unclear whether this surface marker pattern is a result of tissue factors encountered in the intestinal epithelium, or is a characteristic of a separate lineage of cells. Like cNK cells, ieILC1s express the transcription factors T-bet and Eomes; however, studies in mice provided evidence that ieILC1s do not develop from a conventional NKP (discussed below in Section Development). Like most other ILC1s, ieILC1s are potent producers of IFN-γ, which they release in response to IL-12 and IL-15, and are likely to contribute not only to immune protection but also to chronic inflammation of the intestinal mucosa.

Murine LP ILC1s share characteristics with cNK cells and ieILC1s, including surface expression of NKp46 and NK1.1. However, unlike the latter two subsets, mouse LP ILC1s express high levels of the IL-7 receptor alpha chain (CD127). Furthermore, LP ILC1s are positive for CD27, Thy1, and c-kit, which are not typically expressed on mature cNK cells, and they are negative for most Ly49 receptors. Unlike cNK cells and ieILC1s, LP ILC1s only express T-bet but not Eomes (20). In human intestinal lamina propria, a CD127^+^ ILC subset with ILC1-like functions has recently been described. These cells lack CD56 and NKp46 but express CD161, a marker also found on some human cNK cells, ILC2s, and ILC3s (18, 21–23). These ILC1s are Eomes negative; subsets of these cells are T-bet positive and produce IFN-γ (18, 24).

### Liver ILC1s

The liver contains cNK cells as well as ILC1s, the two of which can be distinguished by their different expression of surface markers and transcription factors. In mice, liver ILC1s are CD3ϵ^−^ NK1.1^+^ DX5^−^ cells with high expression of both the integrin CD49a (VLA-1) and the cytotoxicity-inducing ligand TRAIL. In contrast, cNK cells in the liver are DX5^+^ CD49a^−^ TRAIL^−^. Both cNK and ILC1s in the liver express T-bet; however, like LP ILC1s, liver ILC1s do not express Eomes. Originally believed to represent immature cNK cells (25), liver-resident ILC1s are now recognized as a separate lineage of group 1 ILCs with different progenitor origin, cell trafficking, and functional capacities than cNK cells (26–28). Liver ILC1s are potent producers not only of IFN-γ but also of additional cytokines such as TNF-α, IL-2, and GM-CSF. Liver-resident ILC1s lack the typical cytotoxic machinery – perforin and granzymes A and B – that are common to cNK cells. In contrast, they express high levels of granzyme C and display potent TRAIL-mediated cytotoxicity (17, 29, 30). Human liver ILC1s have been identified as CD56^high^ CD16^−^ cells capable of producing IFN-γ upon activation (31). While the majority of these cells are CD49a^−^ Eomes^+^, a minor subset expresses CD49a^+^ and lacks Eomes, and may thus represent the human counterpart of murine liver CD49a^+^ ILC1s (32). In mice, liver ILC1s are capable of generating immunologic memory to haptens and viral antigens and are capable of mediating robust recall responses upon rechallenge with the same antigen (16, 33), a feature that has not yet been demonstrated for any of the other known ILC1 subsets.

### Salivary Gland ILC1s

In mice, a unique subset of NK-like cells resides in salivary glands (34–36). While these cells closely resemble cNK cells in both surface receptor and transcription factor expression, they also share several features with unconventional NK cells, and in particular, with liver ILC1s. Most notably, both salivary gland ILC1s and liver ILC1s express TRAIL and CD49a (34, 35). Unlike liver ILC1s; however, salivary gland ILC1s express DX5 and Eomes. Salivary gland ILC1s are similar to liver ILC1s in their ability to induce TRAIL-mediated cytotoxicity; however, in contrast to most other ILC1s, salivary gland ILC1s are poor producers of IFN-γ and thus do not entirely fit the classical definition of group 1 ILCs (34, 36).

### ILC1s in Other Tissues

NK-like cells are also found in other non-lymphoid organs, such as the uterus, kidney, and skin of mice and humans (17, 37–41). These cells vary in their phenotype, transcription factor expression, and functions, which may in part be dictated by their specific tissue microenvironment. Common features of these NK-like cells are their relatively poor cytotoxic potential but potent production of cytokines and growth factors. In the pregnant uterus, non-classical NK cells are thought to play important roles during fetal implantation and vascular remodeling at the decidua (40–42). These uterine NK cells share features with cNK cells, such as the expression of certain activating and inhibitory NK cell receptors, and the expression of perforin and granzymes. However, their developmental origin is still debated (40, 41). In the uterus of virgin mice, several distinct cell populations with NK-like (NK1.1^+^ NKp46^+^ T-bet^+^) phenotype were recently described: DX5^+^ CD49a^−^ cells that likely represent cNK cells as well as a DX5^−^ CD49a^+^ putative ILC1 population that contains both Eomes^+^ and Eomes^−^ subsets (17, 37). Further studies are needed to investigate the lineage relationships of these distinct uterine group 1 ILC subsets and their individual functions at this tissue site. DX5^−^ CD49a^+^ ILC1s with similarity to liver ILC1s were also described in murine skin and kidneys (17, 38). In the murine lung, cNK cells have been studied extensively (5); however, it is currently unknown whether lung tissue also contains distinct ILC1 subsets. In human lung, a putative ILC1 subset has recently been identified (43); however, further studies are required to confirm its specific phenotype and to investigate its functions.

### ILC Plasticity

Further complicating the identification of ILC1s, a subset of group 3 ILCs in murine intestinal tissues, termed NCR^+^ ILC3s, was recently shown to convert to IFN-γ-producing, T-bet-expressing cells during microbial infections (20, 21, 44). Conversion of these ILC3s is accompanied by their downregulation of their specific transcription factor RORγt, resulting in a cell population that resembles ILC1s in their phenotype and cellular functions. Due to their different lineage relationship compared to ILC1s, these converted ILC3s, termed “ex-RORγt ILCs,” are not typically regarded as a “true” ILC1 subset. Additional ILC plasticity was recently identified for a subset of human tonsil ILC1s, which in culture with ILC3-polarizing cytokines acquired RORγt expression and switched their cytokine profile from predominantly IFN-γ to the ILC3-specific cytokine IL-22 (24). This conversion was not seen in mice, as LP ILC1s were unable to convert to ILC3s; however, “ex-RORγt ILCs” were capable of reverting back to RORγt-expressing ILC3s (24).

### ILC1 Signature Genes

Transcriptional profiling of ILC1s and cNK cells from liver, small intestine, and spleen recently demonstrated that the gene expression by these tissue ILC1s clearly differs from that of cNK cells. Principle-component analysis from this study placed the tested ILC1 subsets closer to each other than to cNK cells from the same tissues. However, in addition to shared gene signature, each ILC1 subset possesses a tissue-specific transcriptional signature (45). In contrast, liver and spleen cNK cell transcriptional profiles were indistinguishable, indicating that cNK cells from different tissues represent a relatively homogeneous population. Despite the differences between individual ILC1 subsets, common signature genes were identified that distinguish ILC1s from cNK cells; among these are higher transcript levels for CD127, the chemokine receptor CXCR6, and, interestingly, for some T cell receptor (TCR) chains, although no TCR chain protein expression was detected either intracellularly or on the cell surface. The latter finding is intriguing as it suggests common transcriptional programs between ILC1s and T cells.

## Development

**Figure 1:**
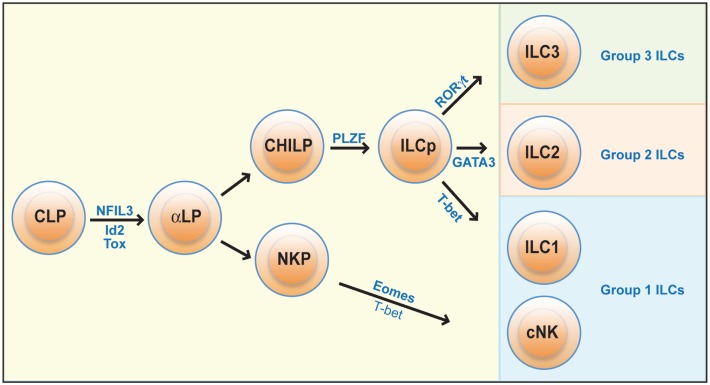
**Development of ILCs**. All ILCs develop from a common lymphoid progenitor (CLP), which differentiate into a committed ILC precursor population (αLP). The development of cNK cells then diverges from that of other ILCs: cNK cells arise from an NK cell precursor (NKP), while all other ILCs are formed from a common helper innate lymphoid precursor (CHILP) that upon upregulation of the transcription factors Id2, PLZF, and GATA-3 become committed innate lymphoid cell precursors (ILCp) and can give rise to ILC1s, as well as to most group 2 and 3 ILCs. Development of mature cNK cells from NKPs is critically dependent on the transcription factors Eomes but also involves T-bet, while ILC1 development from ILCp is dependent on T-bet but not Eomes.

All ILCs develop from a common lymphoid progenitor (CLP), which further differentiates into a shared ILC precursor, termed α-lymphoid precursor (αLP) (or common ILC progenitor, CILP). This shared precursor can be identified by its expression of the integrin α4β7 and of CD127. Further differentiation of αLP generates at least two different precursors with more restricted lineage repopulation capacity: a common helper ILC precursor (CHILP) that can give rise to ILC1s and all other ILC subsets except cNK cells, and an NKP, which can differentiate into cNK cells but no other ILCs (2, 46–48) (Figure 1). Differentiation of these precursors from CLP requires the acquisition of the transcription factors Id2, NFIL3, and Tox; mice deficient in any of these factors show greatly reduced numbers of all ILCs (49–55). Additionally, the Runx family of transcription factors and in particular, Runx3 is required for normal development of group 1 and group 3 ILCs (56, 57). The development of mature ILC1s and cNKs from CHILPs and NKP, respectively, depends on a variety of additional transcription factors and cytokines, many of which have only very recently been elucidated. The following sections discuss the individual requirements for the differentiation of cNK cells and ILC1s from these early precursors.

### Development of cNK Cells

Direct precursors to cNK cells (NKP) were originally defined as lineage-negative (Lin^−^) cells that express the IL-2 receptor beta chain (CD122) but none of the typical NK cell receptors. This precursor population, however, was later found to include cells with T cell differentiation potential and was subsequently refined to include only Lin^−^CD122^+^ cells that co-expressed CD244, CD27, and CD127. The generation of NKPs from CLPs is dependent on the transcription factors NFIL3, Id2, and Tox, which are also involved in the development of CHILPs for ILC development (1, 2). Unlike other ILCs, however, development of cNK cells does not include a precursor that expresses the transcription factor PLZF. As recent studies demonstrated, PLZF is specifically expressed during ILC differentiation from CHILPs, allowing a further refinement of the differences between NKP and ILC1 precursors. PLZF lineage-tracing experiments revealed that true NKPs have the phenotype Lin^−^CD122^+^CD244^+^CD27^+^CD127^+^PLZF^−^α4β7^−^, while ILC1 precursors are characterized by additional expression of PLZF and α4β7 (58).

Further differentiation of NKPs gives rise to immature cNK cells, a process that is accompanied by gradual acquisition of the NK cell receptors NK1.1 and NKp46 and the transcription factors T-bet and Eomes. Immature cNK cells can be identified as DX5^−^ CD27^+^ CD11b^−^ cells, which are T-bet^+^ but Eomes^−^ (23, 25, 59). With these marker characteristics, immature cNK cells have striking similarity to ILC1s, making it difficult to differentiate between the two populations. Maturation of cNK cells is dependent on the cytokine IL-15 and is accompanied by the acquisition of DX5, upregulation of CD11b, and loss of CD27 expression. The cytokine IL-7, which is crucial for the development of group 2 and 3 ILCs, is not required for mature cNK cell or ILC1 development (20, 26, 51). cNK cell maturation is dependent on Eomes and T-bet, as mature cNK cells are absent in Eomes-deficient mice and reduced in numbers and with less mature phenotype in T-bet-deficient mice (25, 60).

### Development of ILC1s

It has been proposed that ILC1s, like group 2 and 3 ILCs, develop from a CHILP that further differentiates into an ILC precursor (ILCp) in a PLZF-dependent process (48, 58). Lineage tracing for PLZF expression revealed that all ILCs, with the exception of cNK cells and a subset of ILC3s (LTi-like ILC3s), develop from a PLZF-positive precursor (48). Among the investigated group 1 ILCs, liver ILC1s and intestinal ieILC1s, but not splenic or liver cNK cells, were lineage traced for PLZF expression, and thus, the former two are likely to arise from the same ILC precursor (48). Other ILC1s such as LP ILC1s or salivary gland ILC1s were not interrogated in this study (48). Interestingly, PLZF-deficient bone marrow can still generate normal numbers of ieILC1s (48), indicating the existence of alternative pathways for ILC1 differentiation, which may involve branching from either PLZF^−^ CHILP or NKP. Within ILCp, the highest ILC1-generating capacity was found in a CD122^+^ subset that lacks the chemokine receptor CXCR5, suggesting that these CD122^+^ CXCR5^+^ ILCp may represent the direct precursors to ILC1s (61).

Differentiation of liver ILC1s and intestinal ILC1s (both LP ILC1s and ieILC1s) further depends on the transcription factor T-bet (17, 19, 20, 25, 62). In contrast to cNK cells, which require Eomes for their final maturation, liver ILC1s and presumably all other Eomes^−^ ILC1s develop independently of this transcription factor (25). Interestingly, there is remarkable diversity among ILC1 subsets in relation to their dependence on other transcription factors and cytokines. Although NFIL3 is required for the development of most group 1, 2, and 3 ILCs, including cNK cells and intestinal ieILC1s, mice deficient in this transcription factor have normal numbers of salivary gland, uterine, and kidney-resident ILC1s (17, 34, 38). In regards to liver ILC1s, the precise role of NFIL3 remains to be determined, as currently conflicting data exists showing either dependence (49, 55) or independence of liver ILC1s on this transcription factor (17, 63). Similarly, dependence on the transcription factor GATA-3 appears to vary between the individual ILC1 subsets. GATA-3 deficiency severely reduces numbers of intestinal LP ILC1s but has no effect on ieILC1 or cNK cell numbers (64). As development of all CD127^+^ group 2 and group 3 ILCs is dependent on GATA-3, it has been suggested that only CD127^+^ ILC1s but not CD127^−^ ILC1s require GATA-3 for their development (20, 64).

Development of most group 1 ILCs, including cNK cells, is dependent on the cytokine IL-15. We recently found that in contrast to all other ILC1s, intestinal ieILC1s were only partially dependent on IL-15, as their numbers were reduced but not absent in mice deficient in the IL-15 receptor alpha chain (19). Although some ILC1 subsets express the IL-7 receptor CD127, IL-7 appears dispensable for ILC1 development, as mice deficient in IL-7 or its receptor CD127 have normal numbers of intestinal LP and liver ILC1s (20, 26, 51).

The differences in transcription factor and cytokine requirements within ILC1s may be a result of different lineage relationships between these individual subsets. Further studies are needed to identify the individual precursors and their location (bone marrow vs. development in peripheral tissues) for each of these ILC1 subsets and elucidate potential pathways of conversion within group 1 ILCs and potentially between other ILCs.

## ILC1s in Antimicrobial Responses

The prototypic function of group 1 ILCs is potent expression of IFN-γ upon activation with cytokines or surface receptor crosslinking. IFN-γ plays important roles in the immune defense to intracellular pathogens, and cNK cells have been recognized for their critical functions in the immune defense against a variety of viral and bacterial pathogens (7, 9). In most of these studies, all cells with NK-like surface phenotype and ability for IFN-γ production were regarded as cNK cells. However, it appears likely that in some of the earlier studies, ILC1s may have contributed to IFN-γ production but were not recognized as a separate lineage. Thus, specific roles of ILC1s, as compared to cNK cells, during immune defenses to pathogens are only now being investigated.

The identification of specific markers for ILC1s, such as CD49a expression in liver or expression of CD127 and lack of Eomes in LP ILC1s, has made it possible to more accurately assess specific host protective roles of ILC1s compared to cNK cells. However, as marker expression can change during cellular activation, results still need to be interpreted with caution. Recent studies have focused on the potential roles of intestinal ILC1s during ­protective responses to intestinal pathogens. New findings in this respect suggest that following oral infection of mice with the intracellular pathogen *Toxoplasma gondii*, ILC1s produce the majority of IFN-γ, as well as TNF-α, while cNK cells and ILC3s contribute to a lesser extent (20). In this particular infection model, Diefenbach and colleagues identified ILC1s as the cell type responsible for controlling infection, which they accomplish by their rapid attraction of inflammatory monocytes to the site of infection. Unfortunately, due to the lack of specific ILC1-knockout mice or depleting antibodies, it is currently impossible to unequivocally attribute a particular function to either ILC1s or cNK cells. In the mentioned study on host responses to *T. gondii*, Diefenbach and colleagues examined T-bet-deficient mice, which completely lack ILC1s but have cNK cells (although in reduced numbers), as a tool to confirm a crucial role of ILC1s during *T. gondii* infection. However, it should be noted that T-bet also plays roles in cNK cell maturation and IFN-γ production (60); thus, the failure of T-bet-deficient mice to control parasite burden may still partially be due to defects in cNK cell functions. Additionally, T-bet is also required for the development of NCR^+^ ILC3s and their conversion into IFN-γ-producing cells (65).

During acute infection with the intestinal pathogen *Clostridium difficile*, ILC1s represent the major innate cell subset to respond with IFN-γ production in the LP, epithelium, and MLN of infected mice (66). Consistent with this finding, Pamer and colleagues further demonstrated that mice lacking ILC1s as well as mice deficient in IFN-γ were more susceptible to lethal *C. difficile* infection. For this study, T-bet-deficient mice on a *Rag*-deficient background were used to deduct a specific role for ILC1s during the immune defense, and adoptive transfer of T-bet^+^ CD127^+^ LP ILC1s to these mice imparted partial protection from lethal infection. However, as ILC1 transfer only provided partial rescue, the authors speculate that the contribution of other innate cells, such as cNK cells, neutrophils and monocytes, is likely required for protective IFN-γ responses to *C. difficile* (66).

IFN-γ is also a major factor in the immune defense to intestinal infections with *Salmonella enterica* serovar *Typhimurium*. A recent study demonstrated that a group of lamina propria ILCs was shown to provide the majority of IFN-γ during acute infection. However, in this study, it was concluded that converted ILC3s, rather than true ILC1s, were responsible for the majority of the produced IFN-γ (65). As illustrated in the examples above, there is emerging evidence that intestinal ILC1s contribute to the immune defense to microbial pathogens. However, to date, an accurate assessment of the individual roles of ILC1s, cNK cells, and converted ILC3 remains impossible. Further exploration of novel markers and transcription factors involved in ILC1 development and functions may aid in further elucidating their specific roles during host protective responses.

## ILC1s in Tissue Inflammation and Autoimmunity

The production of pro-inflammatory cytokines by group 1 ILCs has important functions during antimicrobial immune responses. However, exaggerated or prolonged cytokine responses can also lead to chronic inflammation and autoimmunity. Several studies demonstrated that inflamed intestinal tissues from patients with Crohn’s disease harbor larger numbers of ILC1s, suggesting a role for ILC1s in inflammatory pathology (18, 19). We recently investigated the role of ILC1s in a mouse model of colitis induced by anti-CD40 injection into Rag-deficient mice. In this model, IFN-γ is known as the major factor driving wasting disease and systemic inflammation (67). We find that in this model of inflammatory bowel disease, intestinal ieILC1s contribute to intestinal pathology through production of IFN-γ (19). Similarly, mice with a human immune system show accumulation of IFN-γ-producing human ILC1s in the inflamed intestine upon challenge with the colitis-inducing agent dextran sodium sulfate (18, 19).

A recent study by Victorino et al. demonstrated that ILC1s ­contribute to organ dysfunction seen in a mouse model of ischemic kidney injury. The authors of this study identified NK1.1^+^ non-T cells as the major culprit that mediate kidney dysfunction following ischemia–reperfusion injury, and found that depletion with anti-NK1.1, which depletes both cNK cells and ILC1s in kidneys, ameliorated disease, whereas anti-asialo-GM1 treatment, which preferentially depletes cNK cells, did not protect from disease (38). The exact mechanism for this ILC1-mediated tissue damage awaits further investigation.

In contrast to the potential detrimental roles of ILC1s described above, ILC1s in the salivary gland appear to play tissue-protective functions during chronic infection. ILC1s at this tissue site show low ability for IFN-γ production; however, they can induce cytotoxicity through their expression of TRAIL. Two recent studies demonstrated that during chronic infection with murine cytomegalovirus, ILC1s protected from autoimmunity by regulating both innate and adaptive immune responses in the salivary gland. In these studies, salivary gland NK1.1^+^ cells were shown to preserve gland functions by limiting eosinophil infiltration and by preventing T cell-mediated autoimmunity through TRAIL-mediated cytotoxicity toward activated CD4 T cells (35, 68). Although individual roles of cNK cells vs. ILC1s were not investigated in these studies, a contribution of salivary gland-resident ILC1s appears likely, in particular in relation to the observed TRAIL-mediated regulatory roles.

## Open Questions

The ILC1 field is still in its infancy, and many questions remain as to their specific developmental pathways, and to the lineage relationship between the to-date characterized ILC1s subsets. For instance, the exact molecular processes that mediate lineage specification of ILC1 vs. cNK cells during the branch point from αLP to either CHILP or NKP are insufficiently understood. Furthermore, for several of the putative ILC1 subsets, a lineage relationship to ILC1s awaits confirmation. Examples of those subsets include salivary gland ILC1s, uterine ILCs, and subsets of dermal ILCs. Additional NK-like cells with non-typical phenotype and functions have been described, such as CD127^+^ blood and splenic ILC1s and GATA-3-dependent thymic NK cells (13, 45, 69, 70).

Furthermore, to date, the extent of ILC plasticity is not clear. As discussed above, ILC3s can convert to ILC1-like cells during inflammatory conditions. A recent study now also provided evidence that a subset of human ILC1s can gain ILC3-like phenotype and function (24). Further studies will be required to define whether this ILC1 subset represents a precursor population that differentiates into ILC3s, or a mature ILC1 population that undergoes conversion to ILC3s, and to investigate the mechanism and functional relevance of this ILC1 to ILC3 conversion.

The roles of ILC1s vs. cNK cells during protective host responses and chronic inflammation are only now starting to being investigated and there is little evidence of their beneficial or pathogenic functions during these processes. Group 2 and 3 ILCs play important roles both as initiators of local inflammation, as well as in the resolution of inflammation and restoration of tissue integrity (71). Further studies are needed to elucidate the specific contributions of ILC1s in these processes, and to investigate potential crosstalk between ILC1s with other ILCs and with adaptive immune cells during the initiation and resolution of inflammation.

## Author Contributions

AF researched the literature and wrote the article.

## Conflict of Interest Statement

The author declares that the research was conducted in the absence of any commercial or financial relationships that could be construed as a potential conflict of interest.

## References

[1] Artis D Spits H. The biology of innate lymphoid cells. Nature (2015) 517(7534):293–301. 10.1038/nature14189 25592534

[2] Diefenbach A Colonna M Koyasu S. Development, differentiation, and diversity of innate lymphoid cells. Immunity (2014) 41(3):354–65. 10.1016/j.immuni.2014.09.005 25238093 PMC4171710

[3] Spits H Artis D Colonna M Diefenbach A Di Santo JP Eberl G Innate lymphoid cells – a proposal for uniform nomenclature. Nat Rev Immunol (2013) 13(2):145–9. 10.1038/nri3365 23348417

[4] Eberl G Colonna M Di Santo JP McKenzie AN. Innate lymphoid cells. Innate lymphoid cells: a new paradigm in immunology. Science (2015) 348(6237):aaa6566. 10.1126/science.aaa6566 25999512 PMC5658207

[5] Fuchs A Colonna M. Natural killer (NK) and NK-like cells at mucosal epithelia: mediators of anti-microbial defense and maintenance of tissue integrity. Eur J Microbiol Immunol (Bp) (2011) 1(4):257–66. 10.1556/EuJMI.1.2011.4.1 24516732 PMC3918128

[6] Vivier E Raulet DH Moretta A Caligiuri MA Zitvogel L Lanier LL Innate or adaptive immunity? The example of natural killer cells. Science (2011) 331(6013):44–9. 10.1126/science.1198687 21212348 PMC3089969

[7] Lodoen MB Lanier LL. Natural killer cells as an initial defense against pathogens. Curr Opin Immunol (2006) 18(4):391–8. 10.1016/j.coi.2006.05.002 16765573 PMC7127478

[8] Sun JC Lanier LL NK cell development, homeostasis and function: parallels with CD8(+) T cells. Nat Rev Immunol (2011) 11(10):645–57. 10.1038/nri3044 21869816 PMC4408539

[9] Vivier E Tomasello E Baratin M Walzer T Ugolini S. Functions of natural killer cells. Nat Immunol (2008) 9(5):503–10. 10.1038/ni1582 18425107

[10] Cichocki F Schlums H Theorell J Tesi B Miller JS Ljunggren HG Diversification and functional specialization of human NK cell subsets. Curr Top Microbiol Immunol (2016) 395:63–93. 10.1007/82_2015_487 26472216

[11] Caligiuri MA. Human natural killer cells. Blood (2008) 112(3):461–9. 10.1182/blood-2007-09-077438 18650461 PMC2481557

[12] Ferlazzo G Thomas D Lin SL Goodman K Morandi B Muller WA The abundant NK cells in human secondary lymphoid tissues require activation to express killer cell Ig-like receptors and become cytolytic. J Immunol (2004) 172(3):1455–62. 10.4049/jimmunol.172.3.1455 14734722

[13] Vosshenrich CA Garcia-Ojeda ME Samson-Villeger SI Pasqualetto V Enault L Richard-Le Goff O A thymic pathway of mouse natural killer cell development characterized by expression of GATA-3 and CD127. Nat Immunol (2006) 7(11):1217–24. 10.1038/ni1395 17013389

[14] O’Sullivan TE Sun JC Lanier LL. Natural killer cell memory. Immunity (2015) 43(4):634–45. 10.1016/j.immuni.2015.09.013 26488815 PMC4621966

[15] Gasteiger G Fan X Dikiy S Lee SY Rudensky AY Tissue residency of innate lymphoid cells in lymphoid and non-lymphoid organs. Science (2015) 350(6263):981–5. 10.1126/science.aac9593 26472762 PMC4720139

[16] Peng H Jiang X Chen Y Sojka DK Wei H Gao X Liver-resident NK cells confer adaptive immunity in skin-contact inflammation. J Clin Invest (2013) 123(4):1444–56. 10.1172/JCI66381 23524967 PMC3613925

[17] Sojka DK Plougastel-Douglas B Yang L Pak-Wittel MA Artyomov MN Ivanova Y Tissue-resident natural killer (NK) cells are cell lineages distinct from thymic and conventional splenic NK cells. Elife (2014) 3:e01659. 10.7554/eLife.01659 24714492 PMC3975579

[18] Bernink JH Peters CP Munneke M te Velde AA Meijer SL Weijer K Human type 1 innate lymphoid cells accumulate in inflamed mucosal tissues. Nat Immunol (2013) 14(3):221–9. 10.1038/ni.2534 23334791

[19] Fuchs A Vermi W Lee JS Lonardi S Gilfillan S Newberry RD Intraepithelial type 1 innate lymphoid cells are a unique subset of IL-12- and IL-15-responsive IFN-gamma-producing cells. Immunity (2013) 38(4):769–81. 10.1016/j.immuni.2013.02.010 23453631 PMC3634355

[20] Klose CS Flach M Mohle L Rogell L Hoyler T Ebert K Differentiation of type 1 ILCs from a common progenitor to all helper-like innate lymphoid cell lineages. Cell (2014) 157(2):340–56. 10.1016/j.cell.2014.03.030 24725403

[21] Cella M Otero K Colonna M. Expansion of human NK-22 cells with IL-7, IL-2, and IL-1beta reveals intrinsic functional plasticity. Proc Natl Acad Sci U S A (2010) 107(24):10961–6. 10.1073/pnas.1005641107 20534450 PMC2890739

[22] Mjosberg JM Trifari S Crellin NK Peters CP van Drunen CM Piet B Human IL-25- and IL-33-responsive type 2 innate lymphoid cells are defined by expression of CRTH2 and CD161. Nat Immunol (2011) 12(11):1055–62. 10.1038/ni.2104 21909091

[23] Yu J Freud AG Caligiuri MA. Location and cellular stages of natural killer cell development. Trends Immunol (2013) 34(12):573–82. 10.1016/j.it.2013.07.005 24055329 PMC3852183

[24] Bernink JH Krabbendam L Germar K de Jong E Gronke K Kofoed-Nielsen M Interleukin-12 and -23 control plasticity of CD127(+) group 1 and group 3 innate lymphoid cells in the intestinal lamina propria. Immunity (2015) 43(1):146–60. 10.1016/j.immuni.2015.06.019 26187413

[25] Gordon SM Chaix J Rupp LJ Wu J Madera S Sun JC The transcription factors T-bet and Eomes control key checkpoints of natural killer cell maturation. Immunity (2012) 36(1):55–67. 10.1016/j.immuni.2011.11.016 22261438 PMC3381976

[26] Daussy C Faure F Mayol K Viel S Gasteiger G Charrier E T-bet and Eomes instruct the development of two distinct natural killer cell lineages in the liver and in the bone marrow. J Exp Med (2014) 211(3):563–77. 10.1084/jem.20131560 24516120 PMC3949572

[27] Peng H Tian Z. Re-examining the origin and function of liver-resident NK cells. Trends Immunol (2015) 36(5):293–9. 10.1016/j.it.2015.03.006 25846402

[28] Sojka DK Tian Z Yokoyama WM. Tissue-resident natural killer cells and their potential diversity. Semin Immunol (2014) 26(2):127–31. 10.1016/j.smim.2014.01.010 24548893 PMC4459495

[29] Takeda K Cretney E Hayakawa Y Ota T Akiba H Ogasawara K TRAIL identifies immature natural killer cells in newborn mice and adult mouse liver. Blood (2005) 105(5):2082–9. 10.1182/blood-2004-08-3262 15536146

[30] Tang L Peng H Zhou J Chen Y Wei H Sun R Differential phenotypic and functional properties of liver-resident NK cells and mucosal ILC1s. J Autoimmun (2016) 67:29–35. 10.1016/j.jaut.2015.09.004 26422992

[31] Hudspeth K Donadon M Cimino M Pontarini E Tentorio P Preti M Human liver-resident CD56/CD16 NK cells are retained within hepatic sinusoids via the engagement of CCR5 and CXCR6 pathways. J Autoimmun (2016) 66:40–50. 10.1016/j.jaut.2015.08.011 26330348 PMC4718768

[32] Marquardt N Beziat V Nystrom S Hengst J Ivarsson MA Kekalainen E Cutting edge: identification and characterization of human intrahepatic CD49a+ NK cells. J Immunol (2015) 194(6):2467–71. 10.4049/jimmunol.1402756 25672754

[33] Paust S Gill HS Wang BZ Flynn MP Moseman EA Senman B Critical role for the chemokine receptor CXCR6 in NK cell-mediated antigen-­specific memory of haptens and viruses. Nat Immunol (2010) 11(12):1127–35. 10.1038/ni.1953 20972432 PMC2982944

[34] Cortez VS Fuchs A Cella M Gilfillan S Colonna M. Cutting edge: salivary gland NK cells develop independently of Nfil3 in steady-state. J Immunol (2014) 192(10):4487–91. 10.4049/jimmunol.1303469 24740507

[35] Schuster IS Wikstrom ME Brizard G Coudert JD Estcourt MJ Manzur M TRAIL+ NK cells control CD4+ T cell responses during chronic viral infection to limit autoimmunity. Immunity (2014) 41(4):646–56. 10.1016/j.immuni.2014.09.013 25367576

[36] Tessmer MS Reilly EC Brossay L. Salivary gland NK cells are phenotypically and functionally unique. PLoS Pathog (2011) 7(1):e1001254. 10.1371/journal.ppat.1001254 21249177 PMC3020929

[37] Doisne JM Balmas E Boulenouar S Gaynor LM Kieckbusch J Gardner L Composition, development, and function of uterine innate lymphoid cells. J Immunol (2015) 195(8):3937–45. 10.4049/jimmunol.1500689 26371244 PMC4592103

[38] Victorino F Sojka DK Brodsky KS McNamee EN Masterson JC Homann D Tissue-resident NK cells mediate ischemic kidney injury and are not depleted by anti-asialo-GM1 antibody. J Immunol (2015) 195(10):4973–85. 10.4049/jimmunol.1500651 26453755 PMC4640895

[39] Le Bouteiller P. Human decidual NK cells: unique and tightly regulated effector functions in healthy and pathogen-infected pregnancies. Front Immunol (2013) 4:404. 10.3389/fimmu.2013.00404 24324468 PMC3839044

[40] Manaster I Mandelboim O. The unique properties of uterine NK cells. Am J Reprod Immunol (2010) 63(6):434–44. 10.1111/j.1600-0897.2009.00794.x 20055791

[41] Moffett A Colucci F. Uterine NK cells: active regulators at the maternal-fetal interface. J Clin Invest (2014) 124(5):1872–9. 10.1172/JCI68107 24789879 PMC4001528

[42] Ratsep MT Felker AM Kay VR Tolusso L Hofmann AP Croy BA. Uterine natural killer cells: supervisors of vasculature construction in early decidua basalis. Reproduction (2015) 149(2):R91–102. 10.1530/REP-14-0271 25342175

[43] De Grove KC Provoost S Verhamme FM Bracke KR Joos GF Maes T Characterization and quantification of innate lymphoid cell subsets in human lung. PLoS One (2016) 11(1):e0145961. 10.1371/journal.pone.0145961 26727464 PMC4699688

[44] Vonarbourg C Mortha A Bui VL Hernandez PP Kiss EA Hoyler T Regulated expression of nuclear receptor RORgammat confers distinct functional fates to NK cell receptor-expressing RORgammat(+) innate lymphocytes. Immunity (2010) 33(5):736–51. 10.1016/j.immuni.2010.10.017 21093318 PMC3042726

[45] Robinette ML Fuchs A Cortez VS Lee JS Wang Y Durum SK Transcriptional programs define molecular characteristics of innate lymphoid cell classes and subsets. Nat Immunol (2015) 16(3):306–17. 10.1038/ni.3094 25621825 PMC4372143

[46] Cortez VS Robinette ML Colonna M. Innate lymphoid cells: new insights into function and development. Curr Opin Immunol (2015) 32:71–7. 10.1016/j.coi.2015.01.004 25615701 PMC4648536

[47] Seillet C Belz GT Huntington ND Development, homeostasis, and heterogeneity of NK cells and ILC1. Curr Top Microbiol Immunol (2016) 395:37–61. 10.1007/82_2015_474 26305047

[48] Constantinides MG McDonald BD Verhoef PA Bendelac A. A committed precursor to innate lymphoid cells. Nature (2014) 508(7496):397–401. 10.1038/nature13047 24509713 PMC4003507

[49] Crotta S Gkioka A Male V Duarte JH Davidson S Nisoli I The transcription factor E4BP4 is not required for extramedullary pathways of NK cell development. J Immunol (2014) 192(6):2677–88. 10.4049/jimmunol.1302765 24534532 PMC3948112

[50] Kamizono S Duncan GS Seidel MG Morimoto A Hamada K Grosveld G Nfil3/E4bp4 is required for the development and maturation of NK cells in vivo. J Exp Med (2009) 206(13):2977–86. 10.1084/jem.20092176 19995955 PMC2806474

[51] Satoh-Takayama N Lesjean-Pottier S Vieira P Sawa S Eberl G Vosshenrich CA IL-7 and IL-15 independently program the differentiation of intestinal CD3-NKp46+ cell subsets from Id2-dependent precursors. J Exp Med (2010) 207(2):273–80. 10.1084/jem.20092029 20142427 PMC2822619

[52] Seehus CR Aliahmad P de la Torre B Iliev ID Spurka L Funari VA The development of innate lymphoid cells requires TOX-dependent generation of a common innate lymphoid cell progenitor. Nat Immunol (2015) 16(6):599–608. 10.1038/ni.3168 25915732 PMC4439271

[53] Xu W Domingues RG Fonseca-Pereira D Ferreira M Ribeiro H Lopez-Lastra S NFIL3 orchestrates the emergence of common helper innate lymphoid cell precursors. Cell Rep (2015) 10(12):2043–54. 10.1016/j.celrep.2015.02.057 25801035

[54] Yokota Y Mansouri A Mori S Sugawara S Adachi S Nishikawa S Development of peripheral lymphoid organs and natural killer cells depends on the helix-loop-helix inhibitor Id2. Nature (1999) 397(6721):702–6. 10.1038/17812 10067894

[55] Yu X Wang Y Deng M Li Y Ruhn KA Zhang CC The basic leucine zipper transcription factor NFIL3 directs the development of a common innate lymphoid cell precursor. Elife (2014) 3:e04406. 10.7554/eLife.04406 25310240 PMC4356142

[56] Ebihara T Song C Ryu SH Plougastel-Douglas B Yang L Levanon D Runx3 specifies lineage commitment of innate lymphoid cells. Nat Immunol (2015) 16(11):1124–33. 10.1038/ni.3272 26414766 PMC4618046

[57] Levanon D Negreanu V Lotem J Bone KR Brenner O Leshkowitz D Transcription factor Runx3 regulates interleukin-15-dependent natural killer cell activation. Mol Cell Biol (2014) 34(6):1158–69. 10.1128/MCB.01202-13 24421391 PMC3958033

[58] Constantinides MG Gudjonson H McDonald BD Ishizuka IE Verhoef PA Dinner AR PLZF expression maps the early stages of ILC1 lineage development. Proc Natl Acad Sci U S A (2015) 112(16):5123–8. 10.1073/pnas.1423244112 25838284 PMC4413309

[59] Yokoyama WM Kim S French AR. The dynamic life of natural killer cells. Annu Rev Immunol (2004) 22:405–29. 10.1146/annurev.immunol.22.012703.104711 15032583

[60] Townsend MJ Weinmann AS Matsuda JL Salomon R Farnham PJ Biron CA T-bet regulates the terminal maturation and homeostasis of NK and Valpha14i NKT cells. Immunity (2004) 20(4):477–94. 10.1016/S1074-7613(04)00076-7 15084276

[61] Ishizuka IE Chea S Gudjonson H Constantinides MG Dinner AR Bendelac A Single-cell analysis defines the divergence between the innate lymphoid cell lineage and lymphoid tissue-inducer cell lineage. Nat Immunol (2016) 17(3):269–76. 10.1038/ni.3344 26779601 PMC4755916

[62] Sciume G Hirahara K Takahashi H Laurence A Villarino AV Singleton KL Distinct requirements for T-bet in gut innate lymphoid cells. J Exp Med (2012) 209(13):2331–8. 10.1084/jem.20122097 23209316 PMC3526352

[63] Seillet C Huntington ND Gangatirkar P Axelsson E Minnich M Brady HJ Differential requirement for Nfil3 during NK cell development. J Immunol (2014) 192(6):2667–76. 10.4049/jimmunol.1302605 24532575

[64] Yagi R Zhong C Northrup DL Yu F Bouladoux N Spencer S The transcription factor GATA3 is critical for the development of all IL-7Ralpha-expressing innate lymphoid cells. Immunity (2014) 40(3):378–88. 10.1016/j.immuni.2014.01.012 24631153 PMC4026797

[65] Klose CS Kiss EA Schwierzeck V Ebert K Hoyler T d’Hargues Y A T-bet gradient controls the fate and function of CCR6-RORgammat+ innate lymphoid cells. Nature (2013) 494(7436):261–5. 10.1038/nature11813 23334414

[66] Abt MC Lewis BB Caballero S Xiong H Carter RA Susac B Innate immune defenses mediated by two ILC subsets are critical for protection against acute *Clostridium difficile* infection. Cell Host Microbe (2015) 18(1):27–37. 10.1016/j.chom.2015.06.011 26159718 PMC4537644

[67] Uhlig HH McKenzie BS Hue S Thompson C Joyce-Shaikh B Stepankova R Differential activity of IL-12 and IL-23 in mucosal and systemic innate immune pathology. Immunity (2006) 25(2):309–18. 10.1016/j.immuni.2006.05.017 16919486

[68] Carroll VA Lundgren A Wei H Sainz S Tung KS Brown MG. Natural killer cells regulate murine cytomegalovirus-induced sialadenitis and salivary gland disease. J Virol (2012) 86(4):2132–42. 10.1128/JVI.06898-11 22156514 PMC3302420

[69] Gasteiger G Hemmers S Bos PD Sun JC Rudensky AY. IL-2-dependent adaptive control of NK cell homeostasis. J Exp Med (2013) 210(6):1179–87. 10.1084/jem.20122571 23650439 PMC3674698

[70] Sitrin J Ring A Garcia KC Benoist C Mathis D. Regulatory T cells control NK cells in an insulitic lesion by depriving them of IL-2. J Exp Med (2013) 210(6):1153–65. 10.1084/jem.20122248 23650440 PMC3674700

[71] Sonnenberg GF Artis D. Innate lymphoid cells in the initiation, regulation and resolution of inflammation. Nat Med (2015) 21(7):698–708. 10.1038/nm.3892 26121198 PMC4869856

